# Journal of Community Hospital Internal Medicine Perspectives Second Anniversary

**DOI:** 10.3402/jchimp.v2i4.20218

**Published:** 2013-01-07

**Authors:** Robert P. Ferguson, Christopher D. Kearney

**Affiliations:** 1 Medstar Union Memorial Hospital

The *Journal of Community Hospital Internal Medicine Perspectives* (JCHIMP) was conceived in 2010. This was done in the context of the rapid expansion of Internet-based journals. The number of Internet journals had increased worldwide, including in the United States. There were a significant number of Internet medical journals published in the United States, but what was lacking was a journal aimed at scholarly activity and community hospital departments of medicine. Community hospitals constitute more than half of the training programs in the United States (1).

Scholarly achievements for community hospitals are required by accreditation organizations. Community hospitals often have obstacles to achieve scholarly requirements. They often lack the resources and traditions of many of the university-based academic centers. In recent years, there has been a decline in the viability of regional medical journals, which had been a vehicle to publish scholarly works of residents from, for example, statewide American College of Physicians Associates scientific meetings. Resident-produced posters and oral presentations lacked a vehicle to follow-up with peer review publications in print journals.

**Figure:**
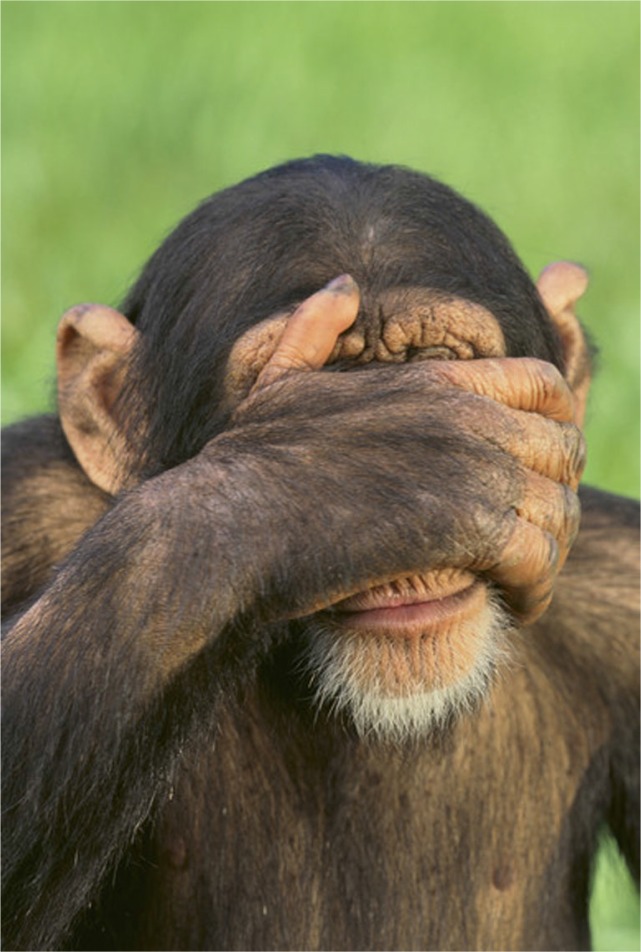
2 year old chimp worried about a possible manuscript rejection. Chimpanzee Picture. Corbis Photos. http://www.fotosearch.com/dgt090/42-17217486/

We took the next step toward a national community hospital journal by developing JCHIMP. We used the technology available to us in open access journals that are characterized by efficiencies of time and costs. Costs are a big issue in any journal, especially one like JCHIMP where no advertising is accepted. We decided that the financing of JCHIMP would take place through donations, modest publication fees—$350 regardless of length (discounted for first author residents and students) and donations from interested parties. The MedStar Union Memorial Department of Medicine Research Fund provided the balance of funding support.

An additional objective of our journal was to include trainees and faculty at community hospitals in the entire process, including peer review. This was a challenge considering a lack of training for students and residents in the journal publication process. The journal relied upon David Solomon as our advisor (2). Dr. Solomon is the experienced editor of the *Journal of Medical Education Online*. Dr. Solomon served not only as a trusted advisor but also as an inspiration in JCHIMP's conception and development. He has served as a guide in the continued evolution of the journal. Upon his recommendation, we have a contract with a publisher, Co-Action Publishing, based in Stockholm, Sweden. This has been an excellent relationship.

Our peer reviewers are largely from the membership of the Association of Program Directors in Internal Medicine (APDIM). Many reviewers are also members of the Community Hospital Education and Research Network (CHERN), an informal association within APDIM. Many of the reviewers were novices at the review process until contacted by JCHIMP. We also encourage resident and medical student peer review involvement when appropriate.

Our general content has evolved over the last 2 years. It includes: perspectives, original research, medical education, case reports, history of medicine, research of EKG cases, and radiologic imaging. We were particularly interested in community hospital editorial perspectives. We hoped the journal would serve as a platform to voice community hospital opinions. Case reports are also a major component of the journal (see below).

One year ago, we summarized the accomplishments of the first year of JCHIMP. During 2011, 50 manuscripts were published on the topics related above. At the end of the first year, our readership included 2,500 ‘unique’ individuals from 88 countries (3). A total of 80 individuals had performed peer reviews using 100% blinded methodology, which assured anonymity for all authors of the 31 published papers. We felt that blinding authorship was an essential element to achieve an unbiased review considering the fact that many journals identify the source of the manuscript and this could lead to less objectivity.

**Figure:**
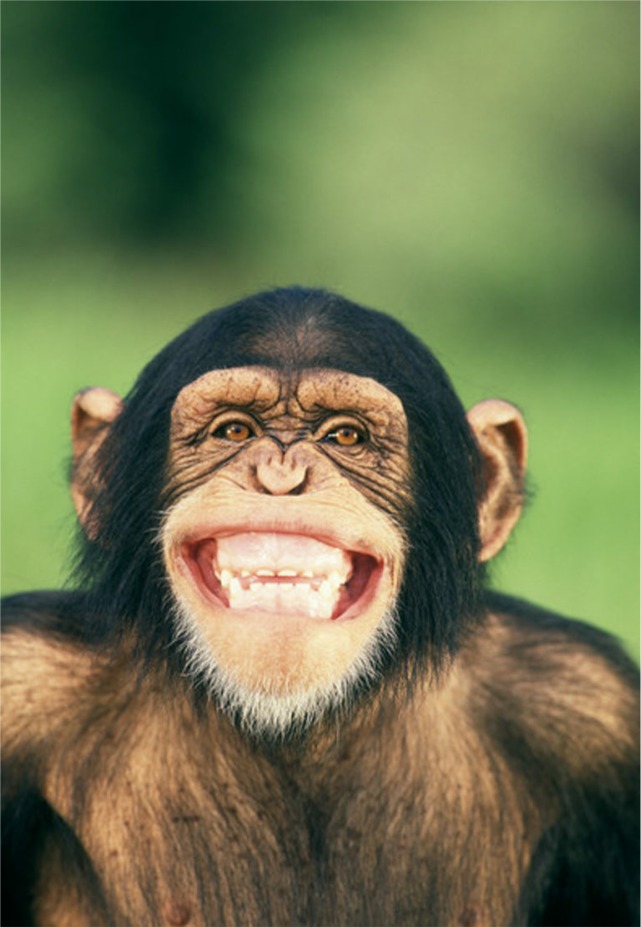
2 year old chimp reacting to a favorable peer review. Grinning Chimpanzee Picture. Corbis Photos. http://www.fotosearch.com/dgt084/42-16477032/

In this second year of the journal, we can list many accomplishments. The total number of manuscripts published in year 2 is 41. The review total in the first 2 years is 136. Active reviewers for the 2012 academic year are listed in Table 1. Highlights of the second year included published perspectives, original research, ECGs and radiology imaging, medical education, and case reports. Despite the limitless potential of the electronic medium with regard to paging, we have maintained our succinctness and encouraged direct and clear papers with reasonable word limits. We feel that case reports are important (4). For a number of reasons, there is a lack of opportunity to publish case reports in most medical journals (5). Case reports can be the scholarly work of residents and students based on their practice-based learning. We encourage submission of unusual but classic cases with an emphasis on historical context such as the meningococcemia case published in the journal earlier this year (6).

**Table 1:** JCHIMP reviewers in 2012

• Carlos Acuna	• Mohammadali Habibi	• Andrew Schuldenfrei
• Chuck Albrecht	• Farnaz Houshmand	• Nawar Shara
• Majd Al Ghatrif	• Ning Jin	• Mansur Shomali
• Richard Alweis	• Paul Kempen	• Peter Sloane
• Donna Astiz	• Maryam Keshtkar Jahromi	• David Gary Smith
• Wayne Campbell	• Bassem Khalil	• Linda Thomas
• Harjit Chahal	• Ramesh Khurana	• David Weisman
• Ehsan Chitsaz	• Jeff Larochelle	• Ashley Wietsma
• Chester Choi	• Mohammad Malik	• Donna Williams
• Dobbin Chow	• Henry Meilman	• Frederick Williams
• John Cmar	• Marita Mike	• Richard Williams
• Janaki Deepak	• Himu Minn	• Amanda Wong
• Melissa Delong	• Dimitra Mitsani	• Radhika Vij
• Stephanie Detterline	• Marc Mugmon	• Alex Yazaji
• Tracey Doering	• Daryn Norwood	• Jeff Zapora
• Norman Dubin	• Venkataraman Palabindala	
• Emmanuel Elueze	• Philip Panzarella	
• Robert Ferguson	• Anne Perieira	
• Colin Franz	• Kathryn Price	
• Ethan Fried	• Ashish Rana	
• Paul Foster	• Zacharia Reagle	
• Steven Gambert	• Farah Salahuddin	

Publishing case reports is one of the missions of the journal. The publishing of a case report and the research required to do it well, including the historical context, is crucial to the development of scholarly attributes of internal medicine trainees. The exercise is a goal unto itself. In this issue, there is an article on Pott's disease secondary to an unusual mycobacterium. The discussion mentions Dr. Percivall Pott and his cohort, Dr. James Paget. Also, looking at imaging of remains from a previous millennium shows that tuberculosis was endemic in the Americas long before it was in Europe (7).

**Fig. 1:**
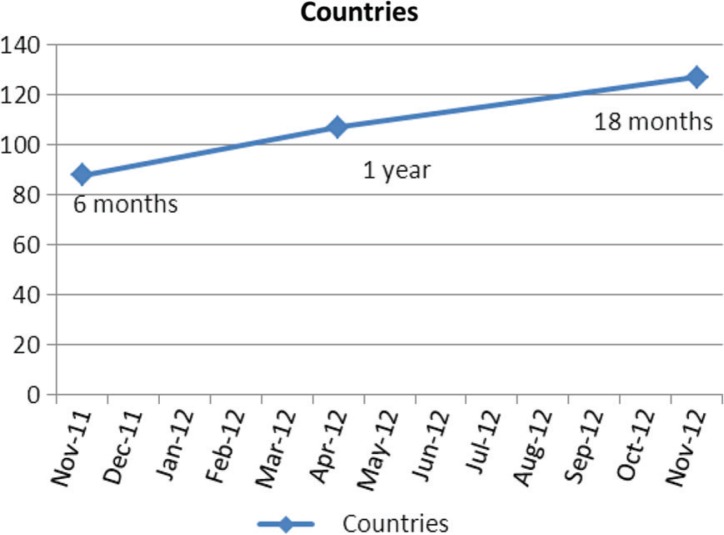
Growth of countries.

**Fig. 2:**
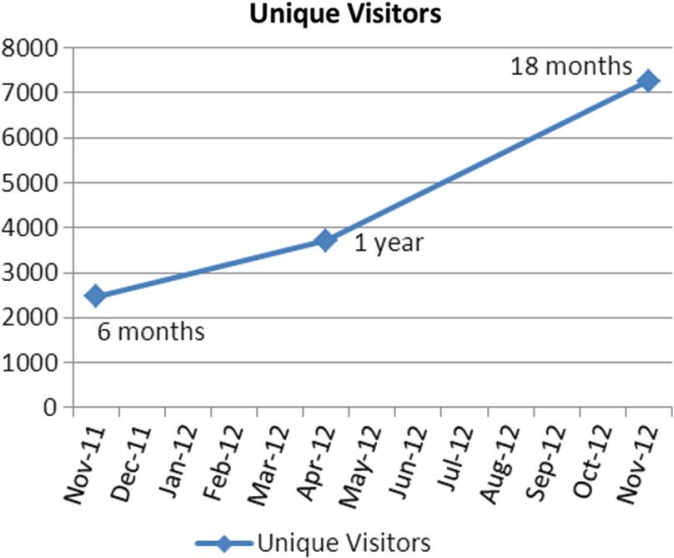
Growth of unique visitors.

It is very encouraging to follow the web data of our journal. Figs. 1 and 2 show the growth curve of readership through 2012. It is remarkable that we can now be read in 127 countries by approximately 7,200 readers. This curve seems to be increasing in slope rather than decreasing and we hope this continues.

The journal has allowed us to introduce faculty, residents, and students to the concept of peer review. We have encouraged peer reviewing by those who are novices in this area. We seek to provide guidance when they need help (8).

Going forward, we would like to stretch our publication schedule pending resources, financial and personal. A semi-monthly publication would be ideal. We believe that the importance of scholarly work will only increase as future residency program accreditations standards demand more scholarly work. We also wanted to maintain a trace of wit as the accompanying photos indicate. We intend to apply for indexing when it is advisable based on publication numbers.

The editor will also continue to highlight the various papers that follow in each issue. Also in this issue are seven manuscripts. There are three research papers: a timely study on the variable effects of medical readmissions by a resident house staff team (9); a literature review with important recommendations by pharmacy faculty on converting morphine to methadone for analgesia (10); and, a survey conducted through the Community Hospital Education and Research Network (www.CHERNINFO.com) Survey Group on methods of teaching residents the nuances of patient health literacy (11). A paper on internal medicine recertification practices and implications for care is the first paper on a CME topic in the two-year history of JCHIMP (12). In addition, there are three case reports: thrombotic thrombocytopenic purpura (TTP) following a medical procedure (13); asymptomatic severe complete heart block in an elderly patient (ECG images) (14); and an old disease (POTTS) in a modern setting with comparisons to similar cases from antiquity secondary to an organism originally detected in an African frog (7).

*Robert P. Ferguson, MD* Chief of Medicine *Christopher D. Kearney, MD* Chief of Geriatrics Medstar Union Memorial Hospital
